# The effect of upper- and lower-body exercise on next-day postprandial triglycerides in healthy young men

**DOI:** 10.3389/fphys.2024.1454731

**Published:** 2024-09-30

**Authors:** Zhentao Zhong, Motohiko Miyachi, Kumpei Tanisawa

**Affiliations:** ^1^ Graduate School of Sport Sciences, Waseda University, Tokorozawa, Japan; ^2^ Faculty of Sport Sciences, Waseda University, Tokorozawa, Japan

**Keywords:** arm-cranking exercise, leg-cycling exercise, moderate to vigorous intensity, postprandial lipaemia, continuous exercise

## Abstract

**Aims:**

High non-fasting triglycerides (TG) concentration is linked to the development of atherosclerosis, and physical activity is commonly recommended to reduce postprandial TG concentration and cardiovascular diseases. Previous studies have demonstrated that acute whole-body (walking and running) or lower-body (leg cycling) aerobic exercise reduces postprandial TG. However, it is unclear whether upper-body exercise (i.e. arm-cranking) with sufficient energy expenditure lowers postprandial TG. Therefore, this study aimed to evaluate the effects of energy-matched upper- and lower-body exercises on postprandial TG concentrations the next day in healthy young men.

**Method and Materials:**

Fifteen healthy young men (age 22.5 ± 1.7 years, height 173.8 ± 5.7 cm, body mass 68.2 ± 8.5 kg, peak oxygen uptake 48.0 ± 5.5 mL/min/kg and physically active) participated in a three-arm crossover trials: 1) arm-cranking, 2) leg-cycling exercise at 70% of mode-specific peak oxygen uptake to induce a net energy expenditure of 1,255 kJ, or 3) rested between 16:00 and 17:00 h on day 1 and consumed two standardised meals for breakfast (10:00 h) and lunch (13:00 h) on day 2. The mean macronutrient content of the breakfast was 44.9 ± 5.6 g fat, 104.8 ± 13.0 g carbohydrate, and 29.4 ± 3.6 g protein, which provided 3.95 ± 0.49 MJ energy (43% fat, 45% carbohydrate, and 12% protein), and that of the lunch was 45.2 ± 5.6 g fat, 106.7 ± 13.2 g carbohydrate, and 33.9 ± 4.2 g protein, which provided 4.06 ± 0.50 MJ energy (42% fat, 44% carbohydrate, and 14% protein).

**Results:**

Time-averaged postprandial serum TG concentrations over 8 h differed among trials (main effect of trial *p* < 0.001) and were lower in the upper- and lower-body exercise trials than in the control trial (1.46 ± 0.54 vs. 1.50 ± 0.69 vs. 1.79 ± 0.83 mmol/L, respectively). The incremental TG area under the curve (AUC) (main effect of trial, *p* = 0.012) was 39% and 37% higher in the control trial than in the upper- and lower-body exercise trials (*p* = 0.025 and *p* = 0.033, respectively). There were no significant differences in incremental TG AUC between the upper- and lower-body exercise trials.

**Conclusion:**

An acute bout of energy-matched upper- and lower-body exercises similarly lowered postprandial TG concentrations the following day in healthy young men.

Trial registration number: UMIN000045449.

Date of registration: 10 September 2021.

## 1 Introduction

Elevated non-fasting triglycerides (TG) are associated with an increased risk of cardiovascular disease (Zilvermist, 1979) and all-cause mortality (Iso. et al., 2014; Nordestgaard. and Varbo, 2014). TG concentrations vary throughout the day in response to consumption of a carbohydrate-rich diet (Riccardi. et al., 2016) and lipid-containing foods (Peddie. et al., 2012). Greater time spent in the postprandial state (Diaz. and Shimbo, 2013; Lira. et al., 2015) lengthens elevations in TG concentrations, reduces high-density lipoprotein cholesterol production, increases low-density lipoprotein cholesterol production (Ford. et al., 2004), and promotes a more atherogenic state (Zilvermist, 1979; Austin. et al., 1990). Therefore, it is important to attenuate non-fasting TG concentrations to reduce the risk of cardiovascular disease.

Physical activity and exercise have been shown to play important roles in cardiometabolic health, and the effectiveness of aerobic exercise in reducing postprandial TG has also been confirmed in many studies (Gill. and Hardman, 2003; Petitt. and Cureton, 2003; Peddie. et al., 2012; Freese. et al., 2014). Moderate-intensity brisk walking, cycling, and running attenuate postprandial TG in healthy men (Ferreira. et al., 2011), obese men (Miyashita, 2008), postmenopausal sedentary women (Murphy. et al., 2000; Kashiwabara. et al., 2018), and adolescent boys (MacEneaney. et al., 2009) when a test meal was served immediately after exercise or 1 day after exercise. For example, Zhang and colleagues (Zhang. et al., 1998) demonstrated that when exercise (1-h running at 60% VO_2max_) was performed 12 h and 1 h prior to the test meal, the postprandial TG area under the curve (AUC) was 51% and 38% lower than that in the control trial, respectively. Thus, ample evidence suggests that acute aerobic exercise is beneficial for attenuating postprandial TG.

Although most of these studies adopted whole-body exercise (i.e. walking and running) or lower-body exercise (i.e. leg cycling) in their experiments and demonstrated a reduction of postprandial TG by these exercises, the effects of upper-body exercise (i.e. arm-cranking) on postprandial TG have not been fully investigated. To date, only four laboratory-based studies have examined the effects of arm-cranking exercise on postprandial TG (Bailey. et al., 2020; Farrow. et al., 2021; Farrow et al., 2022; McMillan. et al., 2021). All of these studies reported no reduction in postprandial TG concentrations after accumulated, moderate-intensity continuous, or high-intensity interval arm-cranking exercise was performed, in adults with paraplegia (Bailey. et al., 2020; Farrow. et al., 2021; McMillan. et al., 2021) and healthy adults (Farrow. et al., 2022). The energy expenditure in these four studies was relatively small (410–619 kJ), which may be the reason for the failure to mitigate postprandial TG concentrations, as energy expenditure appears to be the primary determinant of the exercise-induced reduction in postprandial TG concentrations with aerobic exercise (Freese. et al., 2014). Therefore, the effect of upper-body exercise on postprandial TG should be re-evaluated using an exercise protocol with sufficient energy expenditure and compared to that of energy-matched lower-body exercise in the same participants. However, few studies have compared the effects of upper-body exercise and other well-established exercise modes, including lower-body exercise, in the same individuals. This is because most of these studies only included patients with paraplegia who could not perform leg exercises or elderly people who may have had difficulty performing upper-body exercise with sufficient intensity and duration to elicit postprandial TG-lowering effects. To our knowledge, only one study compared the effect of arm sprint-interval exercise on metabolic response (including plasma TG concentrations) to those of leg sprint-interval exercise and continuous cycling exercise within the same participants and reported that TG concentration 22 h after exercise was not different between these conditions (Francois. et al., 2017). However, this study did not use a high-fat test meal to induce postprandial lipaemia and did not measure TG concentrations during the postprandial period after consumption of the test meal. Therefore, it is necessary to design an experiment using healthy young men to appropriately compare the effects of upper- and lower-body exercises on postprandial TG.

This study aimed to compare the effects of energy-matched upper- and lower-body exercises on postprandial TG concentrations the next day in healthy young men. We hypothesised that energy-matched upper- and lower-body exercises would similarly attenuate postprandial TG concentrations the following day.

## 2 Materials and methods

### 2.1 Participants

This study included three laboratory-based experimental trials (upper-body exercise, lower-body exercise, and control trials) in a random order that were approved by the Ethics Committee on Human Research of Waseda University (approval numbers: 2021-210). Participants provided written informed consent after receiving an explanation of the procedures and risks involved in participating in the study. The participants of the present study were recruited from September 2021 to March 2022 through mailouts placed on posters and flyers on the campus of Waseda University. The experiment was registered in advance with the University Hospital Medical Information Network Center, a system for registering clinical trials (ID: UMIN000045449).

The inclusion criteria were as follows: physically active (leisure-time physical activity: ≥150 min of moderate-intensity physical activity per week or ≥75 min of vigorous-intensity physical activity per week or an equivalent combination of moderate- and vigorous-intensity physical activity as assessed using the International Physical Activity Questionnaire), aged between 20 and 29 years, BMI < 25 and had stable body mass for at least 3 months (±3 kg) before taking part in the study. The exclusion criteria were as follows: current smoking, history of cardiovascular disease or type-2 diabetes, taking medications known to affect study outcomes, or allergies to food items used in the test meals.

Nineteen participants were screened through telephone and emails for eligibility, and one participant was excluded due to smoking history. Eighteen participants visited the laboratory for screening in person, and one participant was excluded because of an allergy to the test meal ingredients. Seventeen participants participated in the main trials, of whom two did not complete the trials owing to failure of blood sampling. Consequently, 15 participants completed all trials.

The study was conducted in accordance with the standards outlined in the Declaration of Helsinki. Only males were involved due to potential sex differences in upper-body exercise capacity which may have impacted the inter-individual differences in fitness level and cause high variability in absolute exercise intensities (Hill. et al., 2019). The physical and physiological characteristics of the participants are listed in Table 1.

**TABLE 1:** Physical and physiological characteristics of the participants.

Characteristic	(n = 15)
Age (years)	22.5 ± 1.7
Height (cm)	173.8 ± 5.7
Body mass (kg)	68.2 ± 8.5
Body mass index (kg/m^2^)	22.5 ± 2.3
Waist circumference (cm)	77.3 ± 5.6
Percent body fat (%)	14.4 ± 5.5
Systolic blood pressure (mmHg)	117 ± 7
Diastolic blood pressure (mmHg)	66 ± 9
Upper-body peak oxygen uptake (ml/kg/min)	35.8 ± 6.9
Lower-body peak oxygen uptake (ml/kg/min)	48.0 ± 5.5
Upper-body maximum heart rate (beats/min)	182.9 ± 13.6
Lower-body maximum heart rate (beats/min)	196.9 ± 12.5
Upper-body peak power output (watts)	161.7 ± 30.0
Lower-body peak power output (watts)	229.6 ± 39.5

### 2.2 Study design and protocol

#### 2.2.1 Preliminary tests

Before the main trials, the participants visited the laboratory on two separate occasions at least 3 days apart. During the first and second visits, participants completed either upper-body exercise or lower-body exercise using an arm-crank ergometer (Ergomedic 881E, Monark Exercise AB, Vansbro, Sweden) or a cycle ergometer (Ergomedic 894E, Monark Exercise AB, Vansbro, Sweden), respectively, at each designated visit to determine the exercise workload at 70% of each participant’s mode-specific peak oxygen uptake (VO_2peak_) and estimate the exercise duration needed to expend a net energy expenditure of 1,255 kJ (300 kcal). The lack of reduction in postprandial TG after acute arm-cranking exercise in the previous studies may have been due to insufficient energy expenditure during exercise (489 ± 146 kJ, 536 ± 100 kJ, and 590 ± 117 kJ, respectively) (Farrow. et al., 2021; Farrow et al., 2022; McMillan. et al., 2021). Hence, we decided to increase net energy expenditure during exercise trials compared to these studies. Based on our pilot studies involving both upper- and lower-body exercises and previous studies reporting a significant reduction in postprandial TG levels after exercise (Miyashita. and Tokuyama, 2008; Miyashita. et al., 2009; Miyashita et al., 2010), we found that exercises with a net energy expenditure of 1,255 kJ were feasible and effective in reducing postprandial TG levels. At both visits, the participants completed two preliminary exercise tests (submaximal and maximal incremental exercise tests) for both upper-body and lower-body exercise trials. A baseline expired air sample was collected before the submaximal incremental exercise test which was designed to determine net energy expenditure during each exercise used in the main trial.

The first exercise test consisted of a 16-min submaximal incremental exercise test using an arm-crank ergometer or a cycle ergometer, the results of which were used to adjust the duration and intensity for the maximal test as well as to determine the exercise workload in the main trials. The initial workload of the submaximal test set at 0.5 kg (30 W) and workload was increased by 0.5 kg (30 W) every 4 min with a constant cadence of 60 rpm for upper-body exercise; or initial 0.5 kg (30 W) was increased by 0.5 –1.5 kg (30–90 W) in the second 4 min stage depending on the participant’s fitness level, followed by 0.5 kg (30 W) increments every 4 min until the last stage with a constant cadence of 60 rpm for lower-body exercise. After a 20-min rest (following completion of the submaximal incremental exercise test), VO_2peak_ was measured directly with an incremental protocol until the participants reached volitional fatigue. The maximal test’s initial workload of the arm-crank and cycle ergometer was set from 1.0 kg (70 W) for arm-cranking exercise and 1.0 kg (70 W) to 2.0 kg (140 W) for cycling exercise depending on the fitness level of each participant and the results of each submaximal incremental exercise test, which was increased by 0.5 kg (35 W) every 3 min with a constant cadence of 70 rpm at the start of each subsequent stage. Heart rate was monitored throughout both preliminary tests with the use of short-range telemetry (Polar A3, Polar Electro, Kempele, Finland) and ratings of perceived exertion (RPE) were assessed periodically in both tests using a 6–20 Borg scale (Borg, 1973). Oxygen uptake, carbon dioxide production, and respiratory exchange ratios were measured continuously throughout both preliminary tests using an online breath-by-breath gas analyser (Quark RMR, COSMED Co. Ltd., Roma, Italy). The regression equation of oxygen uptake on workload calculated from the submaximal test and the mode-specific VO_2peak_ obtained by the maximal test were used to determine the oxygen uptake at 70% of mode-specific VO_2peak_ and the corresponding exercise workload in that intensity, which were used for the main trials. The mean mode-specific VO_2peak_, heart rate max, and peak power output are shown in Table 1.

#### 2.2.2 Standardisation of diet and physical activity

At the end of the last preliminary test, a digital scale was provided to the participants and a professional dietician instructed participants to weigh the food and record their cooking method. On day 1, the participants were asked to bring their breakfast and lunch to the laboratory, which imitated the food that they usually consumed. The researcher and dietician photographed their food and recorded all items and cooking methods on a sheet. The participants were asked to record their dinner at home (take photos, record the cooking method, weigh and record the quantity of ingredients) using the same cooking method as for breakfast and lunch then report details of cooking to the research team by email. On day 2, the researcher and dietician double-checked and recorded details of the dinners provided by the participants. All the recorded information (e.g. food record sheet with photos) of the first main trial was returned to the participants, and they replicated their diet of the first trial in all subsequent trials to standardise the diet on day 1 across trials. Food diaries were analysed by a registered dietician to determine energy intake and macronutrient content. In addition, the participants were asked to remain inactive for 2 days (day 0 and day 1) before each main trial. Participants were also asked to wear a uniaxial accelerometer (Lifecoder-EX, Suzuken Co. Ltd., Nagoya, Japan) to objectively monitor their activity during this period. The accelerometer defined 11 levels of activity intensity (0, 0.5, and 1–9), with 0 indicating the lowest intensity and nine indicating the highest intensity. Level 4 corresponds to an intensity of approximately three metabolic equivalents (Kumahara. et al., 2004). In addition, the total step count (steps/day) was recorded and calculated from the accelerometer using computerised software (Lifelyzer 05 Coach, Suzuken Co. Ltd., Nagoya, Japan). Participants removed the accelerometer during the exercise trial because the purpose of wearing the accelerometer on day 1 was to measure daily physical activity and to ensure that the participants did not exercise before the experiments.

#### 2.2.3 Experimental design

This study comprised two different modes of acute exercise and rest which advanced our understanding of postprandial metabolic responses. The primary outcome of the present study was the postprandial TG concentration over the observational period. The lead investigator generated the randomisation sequence using computer-generated random numbers. The interval between trials was at least 6 days (average 6.98 ± 0.09 days).

Fifteen participants completed three (upper-body exercise, lower-body exercise, and control), 2-day (8 h each day) laboratory-based trials. On the first day of each trial, the participants reported to the laboratory at 09:00 h after an at–least 12-h overnight fast and were asked to bring a packed breakfast and lunch. The participants consumed the packed breakfast and lunch after arrival at the laboratory (09:30–10:30 h) and midway through the day (12:00–13:00 h), respectively. We did not prepare the test meal on day 1 because we tried to perform the experiments in a real-life dietary setting. The participants were asked to record and consume identical amounts of the same food and drink before each of the remaining trials. In each trial, they were asked to rest until 15:30 h in a relaxed state such as read books or used laptops. At 15:30, baseline expired gas samples were collected continuously for 20 min using an online breath-by-breath gas analyser (Quark RMR, COSMED Co. Ltd., Roma, Italy). Between 16:00 and 17:00 h during the upper- and lower-body exercise trials, the participants performed the upper-body exercise or lower-body exercise at a workload predicted to elicit 70% of mode-specific VO_2peak_ for a pre-determined exercise duration which was designed to induce a net energy expenditure of 1,255 kJ (300 kcal). For both exercise trials, the exercise was completed at 17:00 h, so that the time interval between the cessation of exercise and consumption of the first test meal (on the following day) was the same (17 h). In the control trial, participants rested for an hour between 16:00 and 17:00. Heart rate and expired gas were continuously measured during the exercise and rest sessions. RPE was measured every 5 min during the exercise. After exercise or rest, subjective feelings of fatigue and muscle soreness were measured using a visual analogue scale. The participants left the laboratory at approximately 17:15 h, and they were instructed to consume an evening meal before 21:00 h and rest for the remainder of the evening.

**FIGURE 1:**
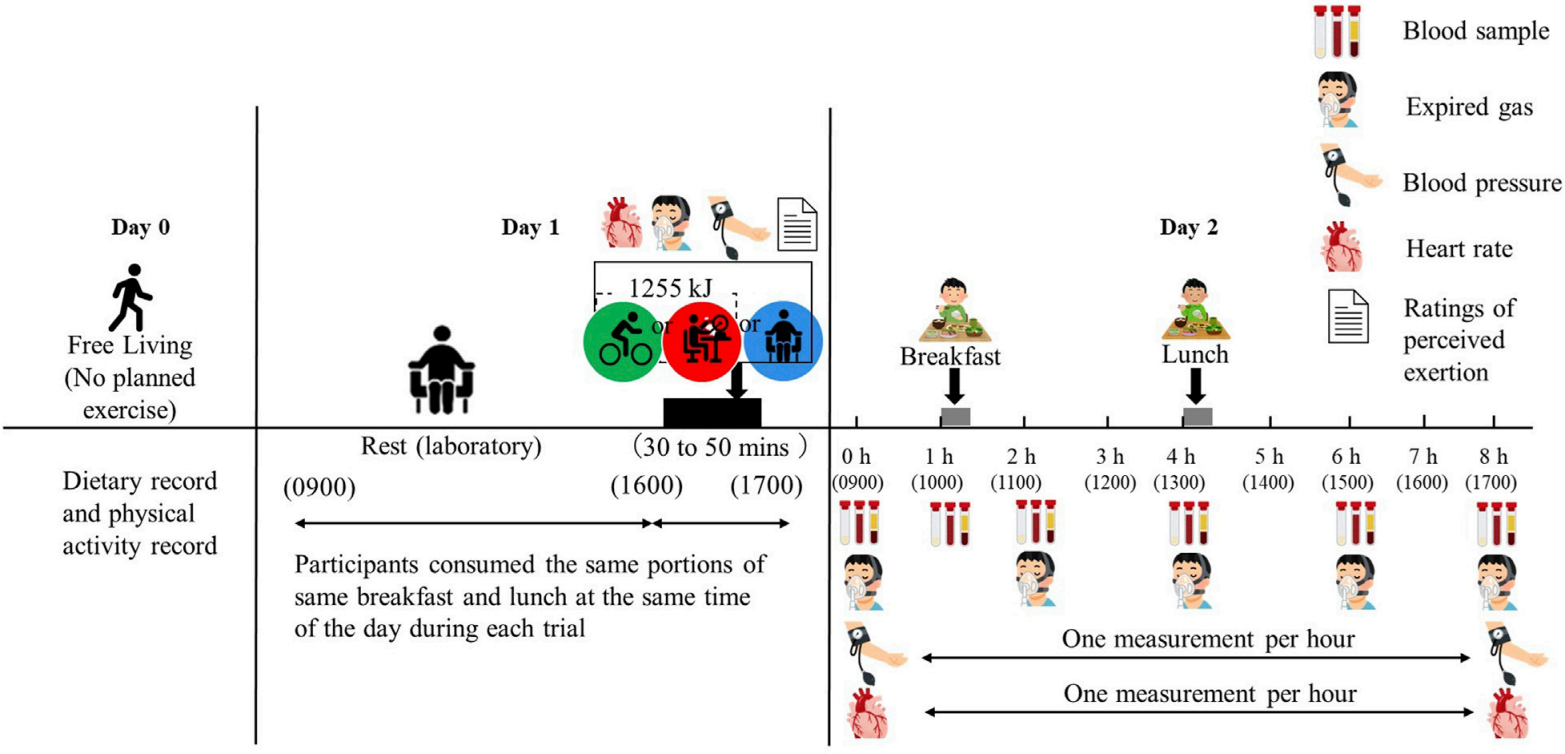
Schematic representation of the study protocol.

On the second day of each trial, the participants reported to the laboratory at 08:45 h after a 12-h overnight fast. The participants sat quietly in a chair for 15 min after their arrival at the laboratory. Baseline venous blood samples were collected via venipuncture at 0 h (09:00 h). A clock was started when the baseline blood samples were collected, and the participants were required to rest in the laboratory for 8 h after the start of baseline blood collection. After a 1-h rest, a second baseline venous blood sample was collected at 1 h (10:00 h). Immediately after blood samples were taken, participants consumed a standardised meal for breakfast at 1 h (10:00 h) (details in “Test meals”). The second test meal was consumed as lunch 3 h after breakfast initiation (13:00 h). Venous blood samples were collected by venipuncture at 2 h (11:00 h), 4 h (13:00 h), 6 h (15:00 h), and 8 h (17:00 h) to measure TG, non-esterified fatty acids (NEFA), 3-hydroxybutyrate (3-OHB), insulin, glucose, and creatine kinase (CK). A schematic illustration of the study protocol for both experiments is shown in Figure 1.

#### 2.2.4 Test meals

The test meal was designed to mimic a typical Japanese meal while increasing fat content as much as possible to induce postprandial lipaemia. Researchers involved in the study and dieticians prepared the test meals. The breakfast consisted of white bread with butter, a salad (made of lettuce and tomato with an Italian dressing), scrambled eggs (an egg with tomato ketchup and soybean oil), soup (made of whole milk with corn soup powder), apples, and yoghurt, which accounted for 38.3% of the total energy intake of day 1. The lunch consisted of a typical Japanese fish dish (made from grilled salmon), a bowl of white rice, soup (made with soybean curd, seaweed, soybean paste, and deep-fried soybean curd), fried vegetables (cabbage and carrot with ham), salad (cucumber, potato, and broccoli with a mayonnaise dressing), and cream crackers, which accounted for 39.4% of the total energy intake of day 1. Energy and macronutrient composition of the test meals are shown in Table 2. The percentage of energy from fat in the test meals was higher than that in the general Japanese population aged between 20 and 39 years (30% ± 8% of total energy from fat reported in the National Health and Nutrition Survey, Japan, in 2019 (MHLW, 2019)). The participants were asked to consume each meal within 30 min, and the consumption time was recorded and replicated in subsequent trials. The mean time to consume breakfast and lunch was 17.7 ± 3.0 min and 17.5 ± 3.2 min, respectively. None of the participants reported nausea or gastrointestinal discomfort during or after the meals. The participants consumed water *ad libitum* during the first trial and the pattern and volume ingested were replicated in subsequent trials. Average water intake was 1,395 ± 526 mL over 8 h.

**TABLE 2:** Energy and macronutrient composition of test meals.

	Breakfast	Lunch
Energy (MJ/kg body mass)	0.0579	0.0595
Energy (MJ)	3.95 ± 0.49	4.06 ± 0.50
Protein (g/kg body mass)	0.43	0.50
Protein (g)	29.4 ± 3.6	33.9 ± 4.2
Protein (% energy)	12.5	14.0
Fat (g/kg body mass)	0.66	0.66
Fat (g)	44.9 ± 5.6	45.2 ± 5.6
Fat (% energy)	42.9	42.0
Carbohydrate (g/kg body mass)	1.54	1.56
Carbohydrate (g)	104.8 ± 13.0	106.7 ± 13.2
Carbohydrate (% energy)	44.6	44.0

### 2.3 Measurements

#### 2.3.1 Anthropometric measurements

Height was measured to the nearest 0.1 cm using a stadiometer (YS-OA, AS ONE Corporation, Osaka, Japan). Body mass was measured to the nearest 0.1 kg using a body composition analyser (MC-780A-N, Tanita Corporation, Tokyo, Japan). Body mass index was calculated as weight in kilograms divided by the square of height in metres. Waist circumference was measured to the nearest 0.1 cm at the level of the umbilicus using flexible plastic tape.

#### 2.3.2 Physiological measurements

At each visit, arterial blood pressure was measured twice in the left arm after 5 min of seated rest using a digital blood pressure monitor (Tango M2, SunTech Medical Inc, Morrisville, United States). Blood pressure measurements in the first preliminary test were performed to ensure that the participants did not have hypertension. Blood pressure measurement in each main trial was performed for safety reasons and were not used for analysis. RPE were assessed periodically (i.e. prior to 16:00 h and then every 5 min until 17:00 h) using a subjective 6–20 scale (Borg, 1973) during a 300 kcal exercise or rest between 16:00 h and 17:00 h. Expired gas samples were collected by an online breath-by-breath gas analyser (Quark RMR, COSMED Co. Ltd., Roma, Italy) for 15 min at 0 h (09:00–09:15 h), 2 h (10:45–11:00 h), 4 h (12:45–13:00 h), 6 h (14:45–15:00 h), and 8 h (16:45–17:00 h) to evaluate resting substrate oxidation rate and energy expenditure. The heart rate was measured using a heart rate monitor (Polar A3, Polar Elctro, Kempele, Finland).

#### 2.3.3 Blood biochemical measurements

For serum TG, NEFA, 3-OHB, and CK measurements, venous blood samples were collected in tubes containing clotting activators to isolate serum (Venoject 2, Terumo Corporation, Tokyo, Japan). Samples were allowed to clot for 30 min at room temperature and then centrifuged at 1861 *g* for 10 min at 4°C. Serum was removed, divided into aliquots, and stored at −80°C for later analysis. For plasma glucose and insulin measurements, venous blood samples were collected in sodium fluoride-ethylenediaminetetraacetic acid (EDTA) and dipotassium salt-EDTA tubes (Venoject 2, Terumo Corporation, Tokyo, Japan), respectively. Both the tubes were immediately centrifuged and treated as described above. Enzymatic, colourimetric assays with automated analysers were used in the determination of serum TG (Pure Auto S TG-N, Sekisui Medical Co. Ltd., Tokyo, Japan), serum NEFA (NEFA-HR, Fujifilm Wako Pure Chemical Industries, Ltd., Osaka, Japan), serum 3-OHB (KAINOS 3-HB, Kainos Laboratories, Inc., Tokyo, Japan), serum CK (CK, Fujifilm Wako Pure Chemical Co., Osaka, Japan), and plasma glucose (GLU-HK(M), Shino-Test Corporation, Tokyo, Japan). Serum plasma insulin levels (Mercodia Insulin ELISA, Mercodia AB, Uppsala, Sweden) were measured using enzyme-linked immunosorbent assays. Samples for each participant were analysed within the same run for each measure. The within-batch coefficients of variation for TG, NEFA, 3-OHB, CK, glucose, and insulin, as determined by running samples in duplicate, were 0.8%, 1.1%, 0.7%, 0.6%, 0.6%, and 7.6%, respectively.

### 2.4 Statistical analyses

We calculated the required sample size based on data from a previous study (Kim. et al., 2014) using G*Power 3.1.9.6 (Fual. et al., 2007). The power calculation was based on the effect size based on a two-group comparison study that examined the effect of cycling exercise on postprandial TG levels in healthy young men because only a small number of studies conducted a three-trial comparison in the above settings and we did not find the effect size (mean and standard deviation) for power calculation from these studies. A previous study reported that prior cycling exercise (15 h before consuming the test meal) at 74% of maximal heart rate for 60 min reduced postprandial TG concentration in nine healthy males (Kim. et al., 2014). This sample size was calculated to detect an effect size of 0.84 (Cohen’s *d*) using a paired *t*-test for comparison between trials. For two trials with an alpha level set at 0.05 and a correlation of 0.5, an estimated total sample size of 14 would achieve 80% power to detect between-trial differences. To confirm that the estimated sample size based on two-group comparison study was reasonable, we additionally performed a power calculation for a three-trial repeated measures one-way analysis of variance assuming a large effect size (Cohen’s f = 0.4). For three trials with an alpha level set at 0.05, and a correlation of 0.5, an estimated total sample size of 12 would achieve 80% power to detect between-trial differences, indicating that the estimated sample size based on the two-trial comparison study was reasonable.

Data were analysed using IBM SPSS Statistics, version 27.0, for Windows (IBM Corporation, New York, NY, United States). The Shapiro–Wilk test was used to check for the normality of distribution, and all parameters were found to be normally distributed. Total and incremental AUC were calculated using GraphPad Prism version 9.2.0 for Windows (GraphPad Software Inc., California, United States). A linear mixed model for repeated measures (time and trial) was used to examine the differences between the three trials for physical activity data, recorded dietary data, physiological response data during exercise, fasting serum or plasma concentration, serum or plasma concentrations over time, total/incremental AUC values, and indirect calorimetry data. Where significant trial-by-time interactions and trial effects were found, the data were subsequently analysed using post hoc analysis and adjusted for multiple comparisons using the Bonferroni method. The 95% confidence intervals (95% CI) for the mean absolute pairwise differences between trials were calculated using the t-distribution and degrees of freedom (*n*-1). Absolute standardised effect sizes (ES) were used to supplement these findings. An ES of 0.2 was considered the minimum significant difference in all outcome measurements, 0.5 moderate, and 0.8 large (Cohen, 1988).

Data are reported as mean ± standard deviation (SD). Statistical significance was set at *P* < 0.050.

## 3 Results

### 3.1 Dietary record and physical activity

Mean self-reported energy intake for the day 1 (eaten breakfast and lunch at laboratory, dinner at home but before 21:00) calculated by dietician to each trial was 10.3 ± 2.5 MJ (2,464 ± 595 kcal). Energy intake equated to 16% ± 6% (81 ± 31 g/day) from protein, 16% ± 8% (80 ± 38 g/day) from fat, and 68% ± 14% (344 ± 73 g/day) from carbohydrate. Body mass did not differ on the morning of each main trial (68.2 ± 8.4 vs. 68.2 ± 8.3 vs. 68.3 ± 8.5 kg for the control, upper-body exercise, and lower-body exercise trials, respectively: ES = 0.010, main effect of trial *p* = 0.436). The individual habitual dietary and physical activity data are shown in Supplementary Table S1.

The step counts recorded on day 0 did not differ among the trials (9,099 ± 3,720 vs. 9,252 ± 4,255 vs. 9,834 ± 5,648 steps/day for the control, upper-body exercise, and lower-body exercise trials, respectively: ES = 0.100, main effect of trial *p* = 0.794). The step counts recorded on day 1 did not differ among the trials (9,715 ± 6,933 vs. 9,016 ± 4,824 vs. 9,909 ± 3,422 steps/day for the control, upper-body exercise, and lower-body exercise trials, respectively: ES = 0.118, main effect of trial *p* = 0.684).

### 3.2 Physiological responses during exercise

The physiological responses during exercise in the upper- and lower-body exercise trials are shown in Table 3. Exercise time was significantly longer in the upper-body exercise trial than in the lower-body exercise trial (*P* = 0.002). Gross energy expenditure did not differ between the upper- and lower-body exercise trials (*P* = 0.198). Power output was significantly higher in the lower-body exercise trial than in the upper-body exercise trial (*P* < 0.001). The rating of perceived exertion was significantly higher in the upper-body exercise trial than that in the lower-body exercise trial (*P* = 0.004). The mean relative exercise intensity, respiratory exchange ratio, heart rate, fat oxidation, and carbohydrate oxidation did not differ (*P* ≥ 0.100) between the upper- and lower-body exercise trials.

**TABLE 3:** Responses to upper- and lower-body exercises.

	Upper-body exercise	Lower-body exercise	P value and effect size
Gross Energy expenditure (kJ)	1424 ± 204	1487 ± 191	*P* = 0.198
Net Energy expenditure (kJ)	1193 ± 204	1299 ± 191	*P* = 0.198
Exercise time (min)	42 ± 8	31 ± 4	* P = 0.002 ES = 1.588
Oxygen uptake (mL/kg/min)	24.4 ± 3.5	34.5 ± 3.1	* P < 0.001 ES = 3.043
Percent maximal oxygen uptake (%)	68 ± 9	72 ± 4	*P* = 0.100
Power output (W)	113 ± 21	161 ± 28	* P < 0.001 ES = 0.618
Ratings of perceived exertion	16 ± 1	14 ± 2	* P = 0.004 ES = 1.197
Heart rate (beats/min)	148 ± 13	151 ± 6	*P* = 0.401
Respiratory exchange ratio	0.91 ± 0.12	0.90 ± 0.04	*P* = 0.590
Total fat oxidation (g)	11 ± 15	12 ± 5	*P* = 0.699
Total carbohydrate oxidation(g)	65 ± 34	63 ± 16	*P* = 0.846

Values are presented as mean ± SD for *n* =15. Means were compared using liner mixed model.

*Significant difference between upper-body exercise and lower-body exercise.

### 3.3 Blood biochemical parameters

#### 3.3.1 Fasting serum/plasma concentrations

The fasting plasma and serum concentrations for each trial are presented in Table 4. No significant main effect of the trial for serum TG, serum 3-OHB, serum NEFA, plasma insulin, plasma glucose concentrations, and serum CK (all *P* ≥ 0.050) was observed.

**TABLE 4:** The fasting concentrations, total area under the curve (tAUC), and incremental area under the curve (iAUC) of serum TG, serum NEFA, serum 3-OHB, plasma insulin, plasma glucose, and serum CK values in the control, upper-body exercise, and lower-body exercise trials.

	Control	Upper-body exercise	Lower-body exercise	Main effect of trial
TG				
Fasting (mmol/L)	0.8 ± 0.3	0.7 ± 0.3	0.7 ± 0.3	*P* = 0.867
tAUC (mmol/L·8 h)	14.2 ± 6.2^a^	11.6 ± 4.1^a^	11.7 ± 5.1	*P* = 0.026
iAUC (mmol/L·8 h)	8.9 ± 4.5^a^ ^,^ ^b^	6.4 ± 2.8^a^	6.5 ± 3.7^b^	*P* = 0.012
3-OHB				
Fasting (mmol/L)	0.03 ± 0.05	0.04 ± 0.04	0.03 ± 0.02	*P* = 0.522
tAUC (mmol/L·8 h)	0.19 ± 0.05	0.20 ± 0.05	0.20 ± 0.04	*P* = 0.680
iAUC (mmol/L·8 h)	0.03 ± 0.38	0.10 ± 0.26	0.02 ± 0.12	*P* = 0.526
NEFA				
Fasting (mmol/L)	0.3 ± 0.1	0.3 ± 0.1	0.3 ± 0.1	*P* = 0.133
tAUC (mmol/L·8 h)	1.9 ± 0.4	2.0 ± 0.6	1.9 ± 0.5	*P* = 0.878
iAUC (mmol/L·8 h)	0.4 ± 1.1	0.4 ± 1.1	0.5 ± 0.9	*P* = 0.155
Insulin				
Fasting (pmol/L)	23 ± 13	23 ± 13	23 ± 19	*P* = 0.991
tAUC (pmol/L·8 h)	1065 ± 546	936 ± 449	904 ± 521	*P* = 0.189
iAUC (pmol/L·8 h)	902 ± 505	773 ± 371	744 ± 398	*P* = 0.153
Glucose				
Fasting (mmol/L)	4.8 ± 0.3	4.7 ± 0.3	4.8 ± 0.3	*P* = 0.294
tAUC (mmol/L·8 h)	31.9 ± 3.6	31.4 ± 2.7	32.2 ± 2.4	*P* = 0.616
iAUC (mmol/L·8 h)	2.1 ± 2.9	2.3 ± 2.5	1.9 ± 1.8	*P* = 0.878
CK				
Fasting (IU/L)	205 ± 117	462 ± 467	235 ± 183	*P* = 0.050
tAUC (IU/L·8 h)	1330 ± 716	2989 ± 3089	1527 ± 1098	*P* = 0.054
iAUC (IU/L·8 h)	105 ± 117	241 ± 240	120 ± 191	*P* = 0.103

Abbreviations: CK, creatine kinase; iAUC, incremental area under the curve; NEFA, non-esterified fatty acids; tAUC, total area under the curve; TG, triglyceride; 3-OHB, 3-hydroxybutyrate.

Values are presented as mean ± SD for *n* = 15. Means were compared using a linear mixed model and post-hoc analysis was adjusted for multiple comparisons using the Bonferroni method. Post-hoc analysis of the main effect of trial:

^a^ *P* < 0.050 between upper-body exercise and control.

^b^ *P* < 0.050 between lower-body exercise and control.

#### 3.3.2 Postprandial serum/plasma concentrations

**FIGURE 2:**
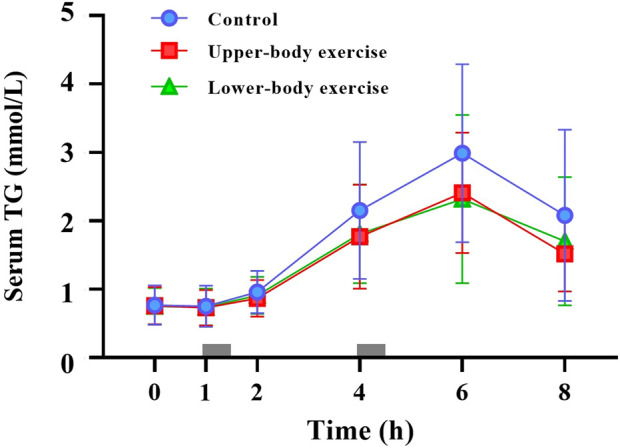
Fasting and postprandial serum triglycerides (TG) concentrations in the control, upper-body exercise, and lower-body exercise trials. Data are presented as the mean ± standard deviation for n = 15. The grey rectangles indicate the times at which the test meals were consumed. Data were analysed using a linear mixed model. Post hoc analysis was adjusted for multiple comparisons using the Bonferroni method. There were significant main effects of trial (*P* < 0.001) and time (*p* < 0.001).

Serum TG concentrations during the 8-h observation period in each trial are shown in Figure 2. The total and incremental TG AUC values for each trial are presented in Table 4. Serum TG concentrations differed between the trials (ES = 0.298, main effect of trial *P* < 0.001, interaction *P* = 0.195). Post hoc analysis revealed that serum TG concentrations were significantly lower in the upper-body and lower-body exercise trials than in the control trial (upper-body exercise vs. control, ES = 0.452, 95% CI –0.54 to −0.11 mmol/L, *P* < 0.001; lower-body exercise vs. control, ES = 0.377, 95% CI –0.51 to −0.08 mmol/L, *P* = 0.004). The total TG AUC (main effect of trial *P* = 0.026) (Table 4) was 23% higher in the control than in the upper-body exercise trial (ES = 0.483, 95% CI –5.23 to −0.03 mmol/L·8h, *P* = 0.047). The incremental TG AUC (main effect of trial, *P* = 0.012) was 39% and 37% higher in the control trial than in the upper-body and lower-body exercise trials, respectively (ES = 0.658, 95% CI –4.64 to −0.25, *P* = 0.025; ES = 0.603, 95% CI –4.55 to −0.15, *P* = 0.033). There were no significant differences in incremental TG AUC between the upper- and lower-body exercise trials.

**FIGURE 3:**
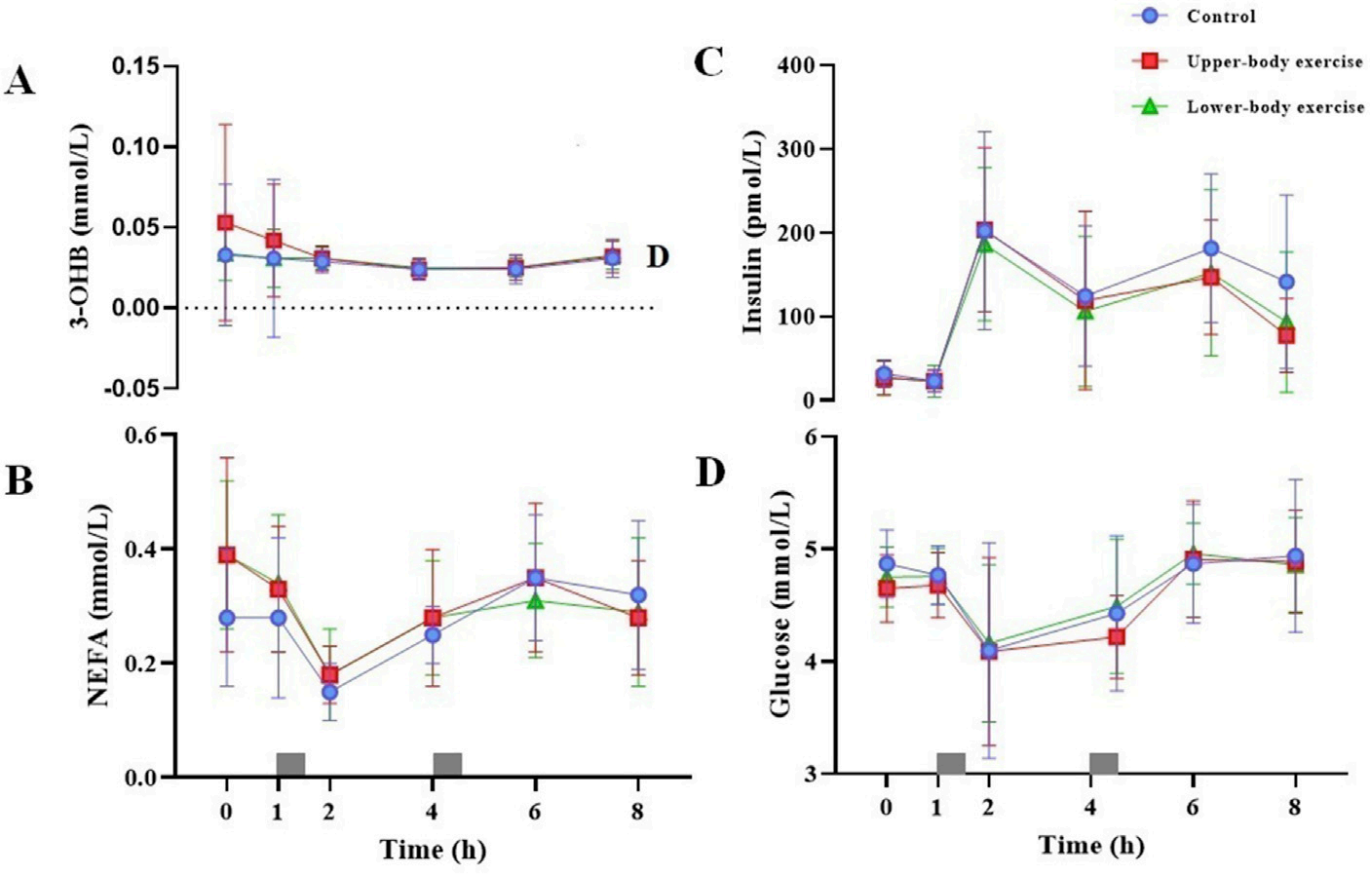
Fasting and postprandial serum 3-OHB **(A)** serum NEFA **(B)** plasma insulin **(C)** and plasma glucose **(D)** concentrations in the control, upper-body exercise, and lower-body exercise trials. Data are presented as the mean ± standard deviation for n = 15. The grey rectangles indicate the times at which the test meals were consumed. Data were analysed using a linear mixed model. 3-OHB, 3-hydroxybutyrate; NEFA, non-esterified fatty acids.

Serum 3-OHB, serum NEFA, plasma insulin, and plasma glucose concentrations during each trial over 8 h are shown in Figure 3. The total and incremental 3-OHB, NEFA, insulin, glucose, and CK AUC are shown in Table 4. Serum 3-OHB, serum NEFA, and plasma glucose concentrations did not differ among the trials (3-OHB, ES = 0.145, main effect of trial *P* = 0.641, interaction *P* = 0.719; NEFA, ES = 0.067, main effect of trial *P* = 0.716, interaction *P* = 0.173; glucose, ES = 0.116, main effect of trial *P* = 0.496, interaction *P* = 0.975). The total and incremental 3-OHB, NEFA, and glucose AUC did not differ among the trials (*P* ≥ 0.155) (Table 4). Plasma insulin concentrations differed among the trials (ES = 0.200, main effect of trial *P* = 0.048, interaction *P* = 0.602). Although the post hoc analysis did not show a significant difference in postprandial plasma insulin concentrations between trials, they tended to be lower in both exercise trials than in the control trial (upper-body exercise vs. control, *P* = 0.124; lower-body exercise vs control, *P* = 0.079). These findings were confirmed by analysing the AUC values for insulin for the entire trial duration (upper-body exercise vs. control, *P* = 0.170; lower-body exercise vs control, *P* = 0.076). Postprandial serum CK concentrations differed among the trials (ES = 0.444, main effect of trial *P* < 0.001, interaction *P* = 1.000). Post hoc analysis revealed that serum CK was significantly higher in the upper-body exercise trial than in the lower-body exercise and control trials (upper-body exercise vs. control, ES = 0.592, 95% CI 145–333 international units per litre [IU/L], *P* < 0.001; upper-body exercise vs. lower-body exercise trial, ES = 0.540, 95% CI 117–305 IU/L, *P* < 0.001). Although the total and incremental CK AUC did not differ among the trials, the values tended to be higher in the upper-body exercise trial than in the lower-body exercise and control trials (Table 4).

### 3.4 Indirect calorimetry

The mean rate of fat oxidation (g/min) on day 2 differed among trials (upper-body exercise, 0.13 ± 0.03 g/min; lower-body exercise, 0.13 ± 0.04 g/min; control, 0.11 ± 0.05 g/min) (ES = 0.298, main effect of trial *P* = 0.018, main effect of time *P* = 0.284, trial-by-time interaction *P* = 0.980). A *post hoc* analysis of the main effect of the trial revealed that the mean rate of fat oxidation was significantly higher in the upper-body exercise trial than in the control trial (ES = 0.459, 95%CI 0.001–0.030, *P* = 0.031). There was no difference in the mean rate of fat oxidation between the lower- and upper-body exercise trials (ES = 0.000, 95%CI -0.016–0.013, *P* = 1.000) or between the control and lower-body exercise trials (ES = 0.436, 95%CI -0.028–0, *P* = 0.061). The mean rate of carbohydrate oxidation (g/min) over the experimental period did not differ (ES = 0.136, main effect of trial *P* = 0.267, main effect of time *P* = 0.963, trial-by-time interaction *P* = 0.705) among the trials (upper-body exercise, 0.09 ± 0.10 g/min; lower-body exercise, 0.09 ± 0.11 g/min; control, 0.11 ± 0.09 g/min). The mean gross energy expenditure (kJ/min) over the experimental period did not differ (ES = 0.178, main effect of trial *P* = 0.099, main effect of time *P* = 0.013, trial-by-time interaction *P* = 0.652) among the trials (upper-body exercise, 6.53 ± 1.17 kJ/min; lower-body exercise, 6.40 ± 1.46 kJ/min; control, 6.19 ± 1.17 kJ/min).

## 4 Discussion

The primary finding of the present study was that the energy-matched upper- and lower-body exercises similarly attenuated next-day postprandial TG concentrations in healthy young men. To the best of our knowledge, this is the first study to directly compare the effects of upper- and lower-body exercises on postprandial TG in the same individuals and to demonstrate that upper-body exercise can reduce postprandial TG.

Our findings regarding the effect of upper-body exercise on postprandial TG were inconsistent with previous studies (Farrow. et al., 2021; Farrow et al., 2022; McMillan. et al., 2021). Two studies (Farrow. et al., 2021; McMillan. et al., 2021) showed that continuous arm-cranking exercise at moderate intensity did not attenuate postprandial TG in individuals with paraplegia, perhaps because of the relatively low energy expenditure (489 ± 146 kJ and 536 ± 100 kJ). Considering that energy expenditure is the primary determinant of the exercise-induced reduction in postprandial TG concentrations with aerobic exercise (Freese. et al., 2014) contributed to postprandial TG reduction in the present study, the low total energy expenditure used in these two studies may explain the lack of attenuation in postprandial TG concentrations. Another previous study (Farrow. et al., 2022) showed that a continuous arm-cranking exercise at moderate intensity performed in the evening prior to the consumption of a test meal did not alter postprandial TG in healthy young adults. Again, it is possible that the energy expenditure (590 ± 117 kJ) in that study was insufficient to reduce postprandial TG in healthy young men (age 28 ± 11 years) and women (age 23 ± 11 years). Compared to these previous studies, the gross energy expenditure during the upper-body exercise in the present study was more than two-fold higher (1,424 ± 204 kJ), which may contribute to the reduction of postprandial TG. Furthermore, the lower-body exercise (1,487 ± 191 kJ) also attenuated postprandial TG in the present study, as reported in previous studies (Herd. et al., 2001; Gill. et al., 2002; Kolifa. et al., 2004; Miyashita. and Tokuyama, 2008). These results suggest that acute aerobic exercise with sufficient energy expenditure attenuates postprandial TG the following day, regardless of involvement of the upper or lower extremities.

The reduction in postprandial TG concentrations observed in the present study after both the upper- and lower-body exercise trials may have been mediated by several mechanisms. One potential explanation for reduced postprandial TG concentrations in exercise trials is the reduced hepatic secretion of very-low-density lipoproteins (VLDL) (Gill. et al., 2001). However, postprandial serum concentrations of NEFA and 3-OHB (a marker for hepatic oxidation) did not differ among the trials in the present study, suggesting that substrate delivery to the liver for TG synthesis and secretion in VLDL was not significantly different among the trials. Alternatively, the lower tendency of postprandial plasma insulin concentrations in both upper- and lower-body exercise trials may have reduced the insulin-mediated inhibition of skeletal muscle lipoprotein lipase (LPL) activity and, therefore, enhanced TG clearance at this site (Seip. et al., 1997).

This study design using healthy young men as participants, while measuring various physiological and biochemical parameters, enabled us not only to clarify the postprandial TG-lowering effects of upper-body exercise, but also to discuss the reason why the postprandial TG response did not differ between upper- and lower-body exercises. In our experiments, the gross energy expenditure (1,424 ± 204 vs. 1,487 ± 191 kJ), heart rate (148 ± 13 vs. 151 ± 6 beats/min), respiratory exchange ratio (0.90 ± 0.10 vs. 0.90 ± 0.04), total fat (10 ± 14 vs. 13 ± 5 g), and carbohydrate oxidation (64 ± 35 vs. 62 ± 16 g) were similar between the upper- and lower-body exercise trials. In addition, on the next day after exercise, there were no significant differences in blood biochemical parameters (i.e. 3-OHB, NEFA, insulin, and glucose, as shown in Figure 3), fat and carbohydrate oxidation rates, and energy expenditure between the exercise trials. Among these physiological and biochemical parameters, the fat oxidation rate on day 2 may be one of the key factors explaining postprandial TG reduction by exercise, as increased fat oxidation has been suggested to elevate the TG hydrolysis rate after a meal. In the present study, the effects of upper- and lower-body exercise trials on fat oxidation rates on day 2 were comparable, although differences between the lower-body exercise and control trials did not reach statistical significance (upper-body exercise trial: ES = 0.459, 95%CI 0.00 to 0.03, *P* = 0.031; lower-body exercise trial: ES = 0.436, 95%CI –0.03 to 0.00, *P* = 0.061). Therefore, a similar effect size for fat oxidation rate on day 2 between the upper- and lower-body exercise trials may explain why the postprandial TG response did not differ between the upper- and lower-body exercise trials. Taken together, these findings suggest that although the muscle groups used in upper- and lower-body exercises were different, similar metabolic and cardiovascular responses during these exercises similarly stimulated LPL activity in exercised muscles for TG uptake, which resulted in no difference in the postprandial TG response to upper- and lower-body exercises.

In contrast to most of the physiological and biochemical parameters showing a similar trend between the upper- and lower-body exercise trials, serum CK levels, an indirect marker of muscle damage, tended to be higher in upper- than lower-body exercise. Greater muscle damage in the upper-body exercise trial was possibly because participants were unfamiliar with upper-body exercises (none of the participants had experienced arm-cranking exercises) which were performed at a relatively high intensity. Although CK levels were higher in upper- than in lower-body exercises, there was no significant difference in the postprandial TG response between these exercise trials. This finding suggests that exercise-induced muscle damage does not affect postprandial TG concentration the following day.

Upper-body exercises have several benefits (Hicks. et al., 2003; Maire. et al., 2004; Francois. et al., 2017). They lower the risk of injury (U.S. Department of Health and Human Services. Physical activity guidelines for Americans. Washington DC’ 2018) in different populations. For healthy individuals, upper-body exercise is a valuable adjunct exercise for whole-body conditioning (Francois. et al., 2017) such as in sports requiring upper-body strength or as a warm-up before resistance training and performing various recreational physical tasks (Sawka, 1986). In addition, three recent upper-body exercise studies (Bailey. et al., 2020; Farrow. et al., 2021; McMillan. et al., 2021) involving individuals with paraplegia demonstrated that upper-body exercise is an accessible exercise prescription for people with disabilities to improve their fundamental ability of locomotion and other activities (Sawka, 1986). However, because the current study recruited only healthy young men, it should be examined whether the upper-body exercise protocol in this study is feasible for those who need to perform this exercise mode for health such as obese, elderly, and paralysed individuals. Therefore, further studies are needed to establish upper-body exercise protocol for various individuals while maintaining the postprandial TG-lowering effect.

This study had several strengths. First, we directly compared the effects of upper- and lower-body exercises on postprandial metabolism and physiological responses in the same participants under rigorously controlled conditions. Furthermore, this study provides an alternative option for larger populations to maintain and/or improve metabolic health. However, our study had certain limitations. First, dietary intake was only collected for 1 day before the experiment, and food intake was instructed by the research team. Therefore, the dietary intake in this study may not reflect the dietary habits of the participants. Given that dietary habits are important determinants of the postprandial TG response, 3–4 days of dietary records are required for a valid and reliable assessment of regular dietary intake in future research. Second, because the current study included only healthy young participants, the generalisability of this study to other populations is limited. Future studies are required to investigate whether upper- and lower-body exercises similarly attenuate postprandial TG in other populations, particularly in groups at high-risk of cardiovascular diseases, such as obese and elderly people with high cholesterol and TG levels. Finally, our study design did not allow us to examine chronic effects of upper-body exercise on postprandial TG levels. A long-term intervention study is needed to provide evidence that upper body exercises play a significant role in preventive medicine.

## 5 Conclusion

In conclusion, the present study demonstrated that energy-matched acute upper- and lower-body exercises lowered postprandial TG concentrations the next day in young men.

## Figures and Tables

**Table:** 

**AUC**	Area under the curve
**CK**	Creatine kinase
**EDTA**	Ethylenediaminetetraacetic acid
**ES**	Effect size
**LPL**	Lipoprotein lipase
**NEFA**	Non-esterified fatty acids
**RPE**	Ratings of perceived exertion
**SD**	Standard deviation
**TG**	Triglycerides
**VLDL**	Very-low density lipoproteins
**VO2peak**	Peak oxygen uptake
**3-OHB**	3-hydroxybutyrate
**95% CI**	95% confidence interval

## Data Availability

The raw data supporting the conclusions of this article will be made available by the authors, without undue reservation.
