# Supplementary material for: The effect of upper- and lower-body exercise on next-day postprandial triglycerides in healthy young men
Source: Front Physiol. 2024 Sep 30;15:1454731. doi: 10.3389/fphys.2024.1454731 (PMC11471655; doi:10.3389/fphys.2024.1454731)
Supplement: Supplementary file 1 [file Table1.DOCX]

| **Supplementary Table S1.** Individual values of total TG AUC, diet, and physical activity in the control, upper-body exercise, and lower-body exercise trials. | | | | | | | | | | | | | | | |  |
| --- | --- | --- | --- | --- | --- | --- | --- | --- | --- | --- | --- | --- | --- | --- | --- | --- |
| Participants ID | 1 | 2 | 3 | 4 | 5 | 6 | 7 | 8 | 9 | 10 | 11 | 12 | 13 | 14 | 15 | |
| Total TG AUC in control trial (mmol/L∙8h) | 6.83 | 10.12 | 21.93 | 18.74 | 8.05 | 16.41 | 24.35 | 14.09 | 19.73 | 11.27 | 5.36 | 12.76 | 10.85 | 26.43 | 17.14 | |
| Total TG AUC in upper-body trial (mmol/L∙8h) | 8.53 | 9.76 | 18.06 | 15.70 | 7.37 | 18.10 | 15.12 | 15.16 | 18.97 | 8.89 | 6.67 | 11.19 | 7.97 | 13.89 | 8.98 | |
| Total TG AUC in lower-body trial (mmol/L∙8h) | 6.11 | 8.98 | 20.06 | 21.08 | 9.56 | 21.90 | 14.98 | 13.52 | 10.73 | 9.60 | 6.51 | 9.99 | 6.54 | 15.84 | 11.19 | |
| Energy intake on day 1 (kcal) | 2253 | 2899 | 2430 | 2978 | 2215 | 2609 | 3246 | 3848 | 2758 | 2206 | 1585 | 1989 | 1939 | 2009 | 2002 | |
| Protein intake on day 1(g) | 56.7 | 67 | 74 | 86 | 61.9 | 76.1 | 102.5 | 144.5 | 91.2 | 88.5 | 57.5 | 142 | 83.5 | 32.4 | 51.8 | |
| Fat intake on day 1 (g) | 72.3 | 135 | 85.3 | 130 | 66.5 | 113.7 | 57.2 | 149 | 91.5 | 62 | 41.3 | 13.6 | 51.3 | 51.3 | 82.9 | |
| Carbohydrate intake on day 1 (g) | 337.8 | 351.7 | 380.7 | 367.3 | 330.2 | 363.4 | 502.3 | 473 | 253.1 | 331.5 | 236.5 | 313 | 292.9 | 354.6 | 265.5 | |
| Energy expenditure on day 1 in control trial (kcal) | 1802 | 2000 | 1990 | 1848 | 1875 | 2350 | 1994 | 1950 | 1958 | 1796 | 2024 | 1881 | 1889 | 1785 | 1659 | |
| Energy expenditure on day 1 in upper-body trial (kcal) | 1945 | 2028 | 1656 | 1904 | 1852 | 2255 | 1852 | 2025 | 2336 | 1858 | 1958 | 1985 | 1798 | 1680 | 1630 | |
| Energy expenditure on day 1 in lower-body trial (kcal) | 1858 | 2115 | 1664 | 1811 | 1908 | 1985 | 2039 | 2311 | 2384 | 1899 | 1965 | 2017 | 1905 | 1701 | 1638 | |
| Energy balance on day 1 in control trial (kcal) | 451 | 899 | 440 | 1130 | 340 | 259 | 1252 | 1898 | 800 | 410 | -439 | 108 | 50 | 224 | 343 | |
| Energy balance on day 1 in upper-body trial (kcal) | 8 | 571 | 474 | 774 | 63 | 54 | 1094 | 1523 | 122 | 48 | -673 | -296 | -159 | 29 | 72 | |
| Energy balance on day 1 in lower-body trial (kcal) | 95 | 484 | 466 | 867 | 7 | 324 | 907 | 1237 | 74 | 7 | -680 | -328 | -266 | 8 | 64 | |
| Steps counts on day 1 in control trial (steps) | 3926 | 10986 | 8603 | 5924 | 11558 | 13817 | 13032 | 8729 | 7917 | 11916 | 4900 | 4461 | 9284 | 10896 | 6381 | |
| Steps counts on day 1 in upper-body trial (steps) | 10848 | 10109 | 2864 | 6271 | 11178 | 12005 | 11216 | 9264 | 14838 | 15194 | 2158 | 7097 | 6318 | 9769 | 5646 | |
| Steps counts on day 1 in lower-body trial (steps) | 5431 | 11636 | 2943 | 3471 | 17194 | 3825 | 20659 | 13817 | 14560 | 15550 | 3185 | 9233 | 9238 | 10455 | 6318 | |
| IPAQ self-reported exercise | Brisk  walking | Jogging tennis | Jogging moving stuff | Cycling  weight training | Swimming  weight training | Swimming  weight training | Swimming  weight  training | Sailing weight  training | Sailing weight  training | Sailing | Body weight training | Weight training | Resistance training | Running | Walking | |
| IPAQ weekly MVPA time (h) | 3.5 | 2.5 | 2.0 | 3.0 | 12.0 | 18.0 | 18.0 | 16.0 | 16.0 | 12.0 | 3.5 | 6.0 | 9.0 | 3.0 | 3.5 | |
| Abbreviations: IPAQ, International Physical Activity Questionnaire; MVPA, moderate to vigorous physical activity; TG, triglyceride. | | | | | | | | | | | | | | | |  |
